# Psyllium supplementation is associated with changes in the fecal microbiota of horses

**DOI:** 10.1186/s13104-020-05305-w

**Published:** 2020-09-29

**Authors:** Michael J. Mienaltowski, Ashley Belt, John D. Henderson, Tannah N. Boyd, Nicole Marter, Elizabeth A. Maga, Edward J. DePeters

**Affiliations:** Department of Animal Science, University of California Davis, 2251 Meyer Hall, One Shields Ave, Davis, CA 95616 USA

**Keywords:** Psyllium, Silica, Fecal microbiota, Acid detergent fiber, Acid detergent insoluble ash, Equine

## Abstract

**Objective:**

Prophylactic supplementation of psyllium husk is recommended to enhance passage of ingested sand from the gastrointestinal tracts of horses. We hypothesized that psyllium supplementation would increase fecal sand passage and favorably alter bacterial populations in the hindgut. Six yearlings and six mature mares were fed a psyllium supplement in the diet daily for seven days. Voluntarily-voided feces were collected over the course of 29 days, prior, during, and after treatment. Feces were analyzed for acid detergent fiber (ADF) and acid detergent insoluble ash analyses. Microbial DNA was also isolated, and the V4 region of the 16S ribosomal RNA gene was PCR-amplified and sequenced using MiSeq technology.

**Results:**

Fecal ADF concentration was greater in adults while silica concentration was greater in yearlings. Mature mare fecal ADF decreased during and just after supplementation but thereafter increased. No changes in silica levels were noted in either group over time. Fecal microbial population phylogenetic diversity was greatest mid-supplementation and lowest at 11 days post-supplementation. Functional profiles of the microbial communities presented some benefits for psyllium supplementation. These findings provide compelling evidence for further detailed studies of prophylactic psyllium supplementation.

## Introduction

Sand enteropathy is a prominent form of colic in which sand accumulates in the large colon of horses living in environments rich in sandy soil. Treatments for sand enteropathy include intravenous or oral fluids and the administration of laxatives like magnesium sulfate, mineral oil, and psyllium to promote the evacuation of sand from a horse’s GIT [1, 2]. Husk from the *Plantago ovata* plant is considered an effective prophylactic dietary supplement as approximately 50–60% of husk mass creates a hydrocolloid gel that increases fecal output which could push sand out of the GIT [3]. Moreover, formulations of psyllium adhere to sand [4]. Psyllium also has intestinal anti-inflammatory and stimulatory gut motility properties in rodents and rabbits [5, 6]. Veterinarians often recommend the supplementation of psyllium because they believe that it, along with other products, could help to ameliorate the symptoms of sand enteropathy [2].

Since horses are hindgut fermenters, GI microbes play essential roles in the catabolism of complex food compounds, particularly fibrolytic bacteria fermenting structural carbohydrates of the plant cell wall of forages [7]. Commensal microbes can promote a healthy GIT and reduce the ability of pathogenic organisms to colonize [7]. While probiotics have been shown to have positive effects on GIT health and performance [8–10], it has yet to be determined how psyllium affects equine GIT microbes. Constipated humans supplemented with psyllium demonstrated increased stool water and significant changes in fecal microbial populations [11].

We examined the effect of psyllium on silica output and GIT microbial populations before, during, and after supplementation. We hypothesized that psyllium supplementation would lead to increased fecal output of silica and to changes in fecal microbial populations. Acid detergent insoluble ash (ADIA) was used to determine silica levels in the feces. ADIA was previously used to accurately measure sand excretion in horse fed sand, psyllium, and/or mineral oil [12]. This strategy differs from previous techniques—large colon radiographic assessments and/or mesh filters separation of sand from feces [1, 2, 13–15]. Radiography is a popular diagnosis tool for sand colic because it provides information about location and severity within the GIT [14, 16]; however, the scope of this study was to understand sand clearance, even before the animals experienced colic, to explore psyllium’s prophylactic potential. Furthermore, we had concerns about the accuracy of the quantitation of sand using flotation and mesh filters. Fecal microbial DNA was isolated for PCR amplification and sequencing of the V4 region of the 16S ribosomal RNA gene to determine represented microbial populations in each sample. All findings were compared by age group and by study day.

## Main text

### Methods

#### Horses, psyllium supplementation, fecal sample collection and storage

Twelve horses—6 yearlings and 6 mature mares (adult age mean 21.5 ± 6.9 years)—were used in this study based on a protocol approved by the university’s Animal Care and Use Committee (Additional file 1. Table S1). Body weights were calculated by averaging weight tape and scale values [17]. Horse diets included wheat hay for yearlings at morning and evening feedings while mares received alfalfa hay at morning feedings and wheat hay at evening feedings. A commercial psyllium product was supplemented with the daily morning ration balancer according to the manufacturer-recommended dosage (312.5 mg/kg body weight) over the course of 7 days. Voluntarily-voided fecal samples were collected over 29 days (Days 0, 3, 7, 9, 11, 14, 21, 28). Days 0 and 3 were pre-supplementation; psyllium supplementation occurred on Days 4 through 10. Fecal samples were collected by taking feces from the upper middle portion of freshly voided manure piles in order to avoid any contamination from ground soil. Fecal samples were stored at − 20 °C until analysis.

#### Dry sample analyses

Fecal samples were dried in a forced-air oven at 55 °C for 8 h to achieve ≥ 85% dry matter. Samples were then ground in a Wiley mill through a 1 mm screen to fine particulates and analyzed using a Fibertec™ FT122/FT121 manual fiber determination system (Foss Analytics, Denmark). ADIA methods were used to determine the effectiveness of psyllium in regards to sand removal by isolating insoluble matter (silica) within the feces [12, 18, 19].

#### Microbial DNA isolation and analyses

PCR-quality DNA was isolated from 84 fecal samples using the ZR Fecal DNA Kit™ (Zymo Research) as previously described [17, 18]. DNA concentration was determined using a NanoDrop UV spectrophotometer (ThermoFisher Scientific). Primers F515 (forward: 5′-GTGCCAGCMGCCGCGGTAA-3′) and R806 (5′-GGACTACHVGGGTWTCTAAT-3′) were used to amplify the V4 domain bacterial 16S rRNA genes, along with a unique barcode on each forward primer for each sample [20–22]. PCR, product purification, and agarose gel electrophoresis visualization were performed as previously described [20]. Combined barcoded libraries were submitted to the University of California Davis Genome Center DNA Technologies Core for 250 bp paired-end sequencing using the Illumina MiSeq platform. Raw sequence data are freely available at the Sequence Read Archive (SRA): Bio Project PRJNA649589. Amplified DNA sequences for each sample were analyzed using QIIME2 (Quantitative Insights Into Microbial Ecology) [23]. Outputs from QIIME2 were then applied to the LEfSe (Linear Discriminant Analysis Effect Size) tool and PICRUSt (Phylogenetic Investigation of Communities by Reconstruction of Unobserved States) software [24, 25] (Additional file 1. Figure S1).

#### Statistical analyses

Fecal nutritional analysis data were analyzed with Graphpad Prism 6.0 (La Jolla, CA, USA) and with *jamovi* [26] with two-way ANOVA (fixed factors: age, day, age-day interaction; dependent variables: percent fiber, percent insoluble ash, PICRUSt prediction) using Tukey-multiple test corrections for contrasts. Taxonomy data were analyzed with Friedman tests (across days within yearlings and within adults) and with Kruskal–Wallis tests (across groups each day), both with Dunn’s multiple comparison tests; significance was assessed *p* < 0.05.

### Results

**Fig. 1:**
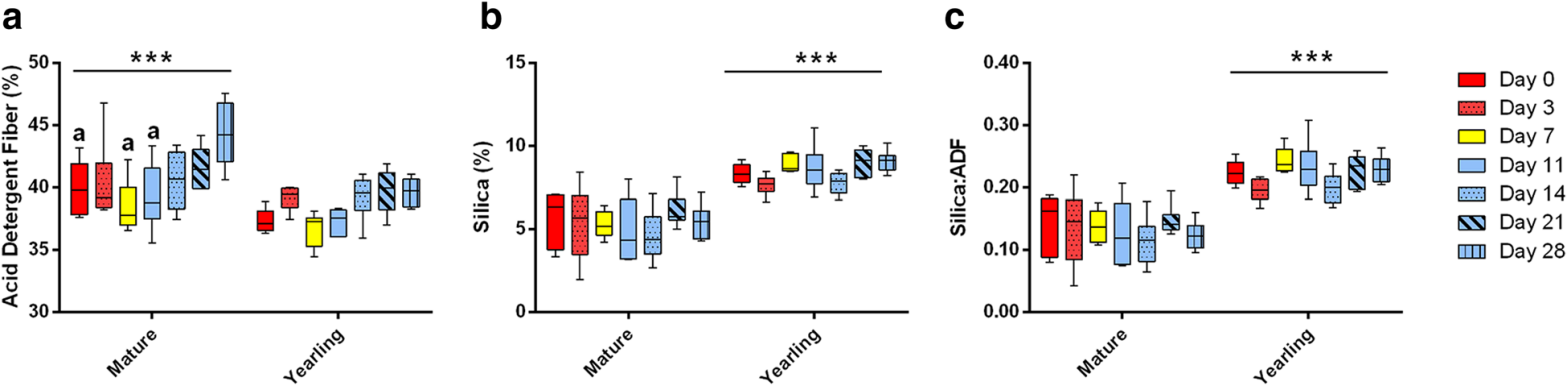
Nutritional Evaluation of Fecal Samples. Overall percent acid detergent fiber (ADF) in feces was greater for mature horses (**a**); for mature horses ADF concentration was lower at Days 0, 7, and 11, relative to Day 28 of the study. Percent silica concentration was comparatively greater overall for yearlings (**b**), even when normalized by ADF in a silica:ADF ratio (**c**). Box color representations are: red, pre-supplementation; yellow, mid-supplementation; blue, post-supplementation. Two-way ANOVA (day, age) analysis was performed with Tukey’s multiple comparison tests for contrasts: a, *p* < 0.05 compared with Day 28; ***p < 0.001

Percent ADF values were greater overall in the adult feces relative to the yearling samples (Fig. 1a). Moreover, Days 0 (pre-supplement) and 7 (mid-supplement) demonstrated lower ADF than Day 28 for mature horses. Fecal ADIA content (silica) was greater in the yearling samples (Fig. 1b), even when comparing silica:ADF ratio (Fig. 1c), yet no significant day-to-day differences were seen in either age group.

QIIME2 was used to analyze diversity of the microbial populations in the samples. Data were rarefied to 4364 reads with all collected samples included. A rarefaction plot revealed abundance of amplification sequence variants (ASVs) found within each group with depths plateauing between 200 and 400 ASVs (Additional file 1. Figure S2a). Faith’s Phylogenetic Diversity—a measure of richness of taxonomical diversity taking into account levels of phylogeny—was greatest on Day 7 for adults and yearlings and at its least on Day 21 for adults and yearlings (Additional file 1. Figure S2b). Shannon H Scores—measurements of evenness of diversity amongst samples—were above 7.0 for all groups and indicated that many species were represented (richness) within similar proportions (evenness) (Additional file 1. Figure S2c). An Unweighted Unifrac PcoA Beta Diversity Plot demonstrates that there was little distinct clustering by day of study or by age (Additional file 1. Figure S2d).

**Fig. 2:**
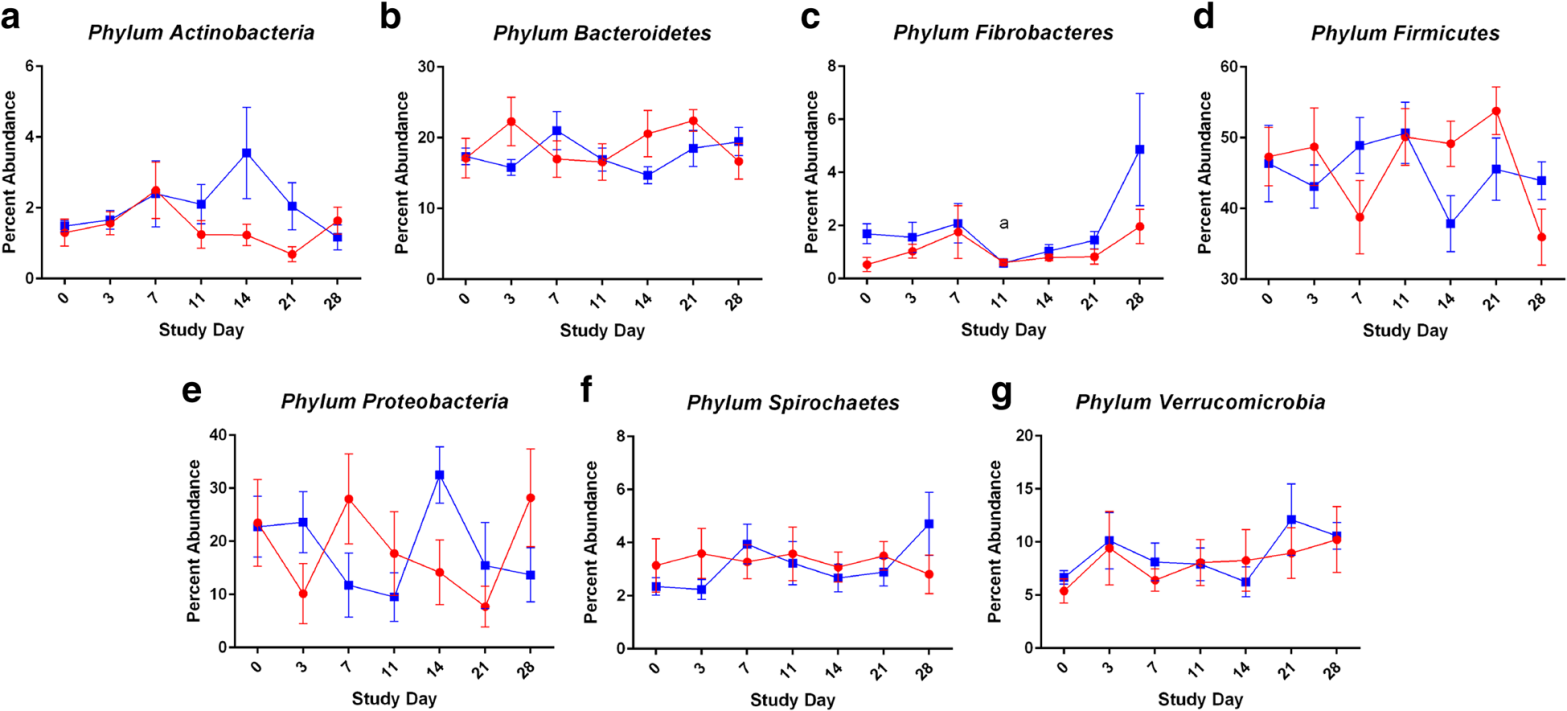
Percent Abundance Plots for Predominant Bacteria. For yearlings (blue) and adults (red), the percent abundance of bacterial populations was plotted as means ± SEM over time for *Phyla Actinobacteria* (**a**), *Bacteroidetes* (**b**), *Fibroacteres* (**c**), *Firmicutes* (**d**), *Proteobacteria* (**e**), *Spirochaetes* (**f**), and *Verrucomicrobia* (**g**). Friedman and Kruskal–Wallis analyses were performed with Dunn’s multiple comparison tests (a, contrast *p*_Dunn’s_ < 0.05 compared with Day 28 within yearlings)

The most abundant phyla included *Firmicutes*, *Proteobacteria*, *Bacteroidetes*, *Verrucomicrobia*, *Fibrobacteres*, *Spirochaetes*, and *Actinobacteria* (Fig. 2). which corroborated findings from our previous study [20]. For phylum *Fibrobacteres*, there was a significant peak at Day 28, roughly 18 days after psyllium supplementation for yearlings (Fig. 2c). Additionally, a dip appeared in bacteria of the phylum *Firmicutes* at Day 14 with a concurrent spike phylum *Proteobacteria* in yearlings (Fig. 2d, e).

Microbial abundance tables at the taxonomical family level were inputted into LEfSe [25]. Several microbial families were identified in yearlings as having significantly changed in at least one time point, including bacteria within families *Methylophilaceae*, *Burkholderiaceae*, *Saprospiraceae*, *Neisseriaceae*, *Fibrobacteraceae*, and *Paraprevotellaceae*; of these, only *Fibrobacteraceae* and *Paraprevotellaceae* families represented abundances greater than 1% (Additional file 1. Table S2). In mature mares, changes were found within bacterial families *Victivallaceae*, *Bacteroidaceae*, *Moraxellaceae*, as well as archaea families *Methanobacteriaceae* and *Methanocorpusculaceae*, with *Victivallaceae*, *Bacteroidaceae*, and *Moraxellaceae* reaching abundances above 1% (Additional file 1. Table S3). Thus, overall LEfSe results demonstrated changes in microbial families with relatively minor differences in abundance levels.

**Fig. 3:**
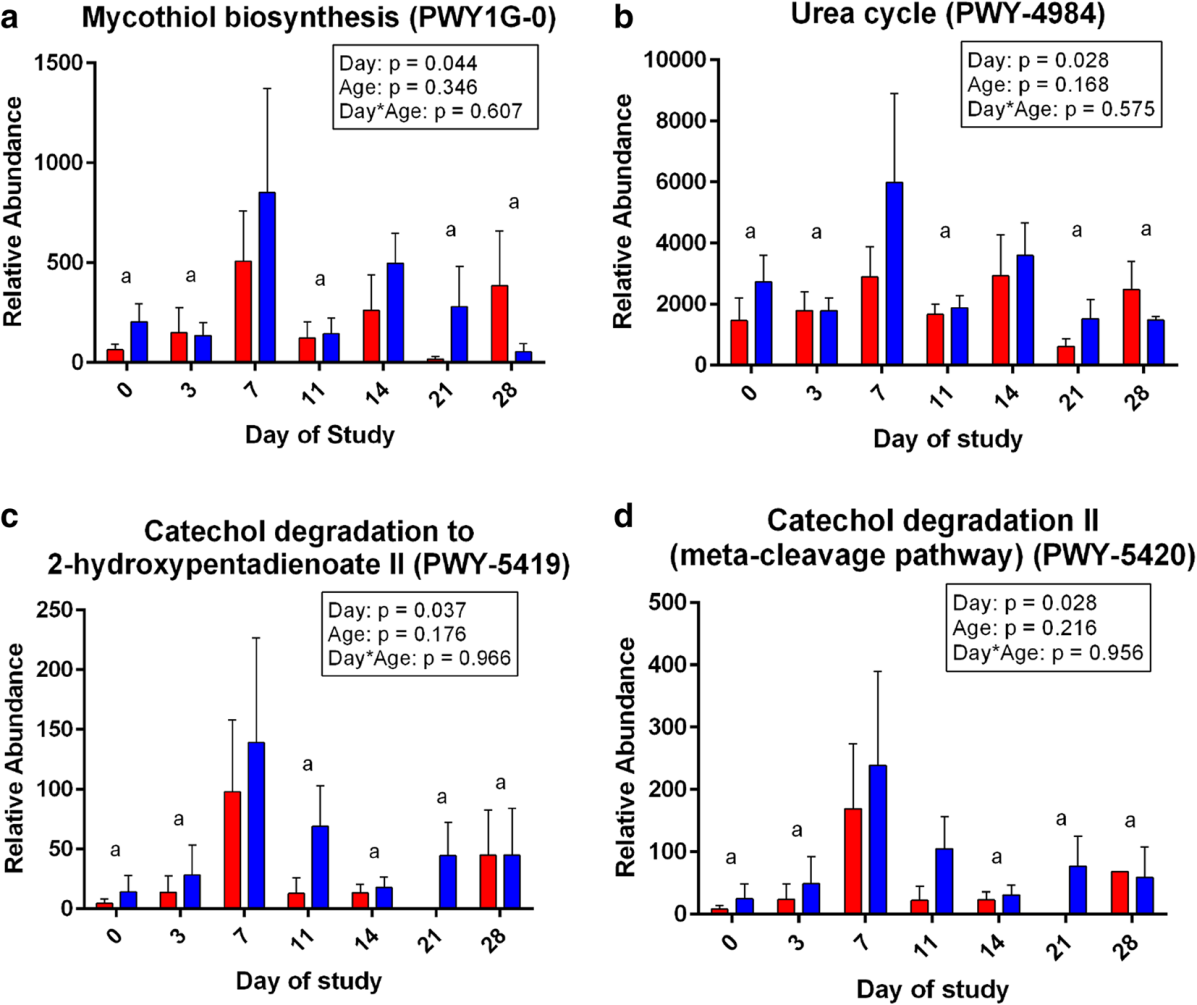
PICRUSt predictions of function. Using PICRUSt analysis of microbiota, increases in mycothiol biosynthesis (**a**), urea cycle activity (**b**), and catechol degradation (**c**, **d**) were found during the period of psyllium supplementation (Day 7). Functional profiling references are from MetaCyc Metabolic Pathway Database. Bars represent mean ± SEM with blue bars representing yearling samples and red bars representing adult samples. Two-way ANOVA indicated *p* < 0.05 for these pathways. Subsequent post-hoc contrasts indicated *p* < 0.05, designated with “a” for comparing those days with Day 7 (mid-supplementation)

Finally, the data were also applied to PICRUSt to infer functional profiles of microbial populations present in the samples [24]. During psyllium supplementation, mycothiol biosynthesis, catechol degradation, and urea cycle activity increased (Fig. 3).

### Discussion

Contrary to our hypothesis, fecal silica analyses (ADIA) from this study did not demonstrate any increase in silica excretion with manufacturer-recommended prophylactic doses of psyllium husk crumbles. Other studies providing therapeutic doses of psyllium as well as combinations of additional therapeutics (prebiotics and probiotics) demonstrated increased clearance of silica with supplementation [2, 13, 15, 27]. We did see greater levels of fecal silica excreted from the yearlings. However, these contrasts could be associated with differences in forages fed to yearlings and adults. Higher relative fecal ADF levels in the mature horses seem to indicate either better forage fermentation in the yearlings, reduced fermentation in the mature mares, or possibly better GIT health in yearlings contributing to more overall sand clearance and greater fermentation.

Microbial populations were analyzed from the standpoint of diversity, populations present, as well as their possible functional roles within the GIT. During and after psyllium supplementation, fecal archaea and bacteria were present in abundances similar to most adult horses [7, 20, 28]. In considering Faith’s Phylogenetic Diversity, the samples with the richest diversity were those collected during mid-supplementation on Day 7. Greater diversity has been positively correlated with a healthy horse GIT; our findings suggest that prophylactic psyllium was beneficial for improving microbial diversity [29, 30]. However, there were no dramatic detectable changes in abundances of microbial populations. Even statistically significant changes in microbial abundances by LEfSe were relatively small with slight reductions in *Burkholderiaceae.* Slight increases were seen in *Fibrobacteraceae* and *Paraprevotellaceae* in yearlings, which contain essential cellulose-degrading bacteria and bacteria found in the GIT of pasture forage-fed horses, respectively [31, 32]. In adults, there was a slight reduction in methanogenic archaea *Methanocorpusculaceae* but slight increases in methanogenic bacteria *Methanobacteriaceae*, which captures hydrogen and improves fermentation efficiency, and bacterial *Moraxellaceae* commonly found in younger horses and foals [20, 30–33]. Increased mycothiol biosynthesis activity in the GIT was demonstrated during psyllium supplementation. Mycothiol is a protective antioxidant produced by bacteria in the Phylum *Actinobacteria* [34]. Moreover, an upturn in urea cycle activity is indicative of increased urea utilization by gut bacteria as a nitrogen source during psyllium supplementation [35]. Finally, catechol degradation pathways were elevated in GIT bacteria at the time of supplementation because psyllium is catechol rich. Furthermore, four days after the last dose of psyllium at Day 14 there was a spike in bacteria from the phyla *Proteobacteria* and a drop in bacteria from the phyla *Firmicutes*; these changes could indicate that after psyllium was no longer present in the GIT, bacterial populations readjusted to gut environment changes. These findings demonstrate that microbial populations were adapting to the psyllium supplied within the GIT. Thus, there is utility to further characterization of the effects of psyllium supplementation on sand excretion and promoting GIT health in horses, particularly at higher levels of supplementation either for prophylaxis or treatment of sand colic [13, 15].

## Limitations

This study does have several limitations. A small number of horses were included (n = 2 groups × 6 horses). Moreover, follow-up studies would benefit from wider representation of various ages of horses. Additionally, more psyllium dosages could have been considered beyond the one manufacturer-recommended prophylactic dose. Furthermore, sand intake was not measured or estimated for the horses during the study, and complete GIT sand quantities excreted were not measured but instead concentrations of ADF and silica in the feces. Thus, total quantities of silica excreted with psyllium supplementation cannot be provided from our measurements. Moreover, the yearlings and mares had differing diets contributing to differences between the forage fiber characteristics in the groups—likely impacting fermentation, the microbial community, and overall gut motility comparisons between yearling and adult groups. We also did not account for breed or sex differences in our analyses. Additionally, we only evaluated microbial populations using 16S amplicon sequencing and did not carry out a metagenomic study which would have had better resolution, thus allowing for distinctions to be found only down to the family level.

## Supplementary information

**Additional file 1: Table S1.** Details on horses from the study. **Table S2.** Yearling LEfSe Findings. **Table S3.** Adult LEfSe findings. **Figure S1.** Microbial DNA next-generation sequencing data analysis methodology. **Figure S2.** Analyses of diversity.

## Data Availability

Data will be available at Sequence Read Archive (SRA): Bio Project PRJNA649589.

## Abbreviations

GIT: Gastrointestinal tract
ADF: Acid detergent fiber
ADIA: Acid detergent insoluble ash
SRA: Sequence Read Archive
QIIME2: Quantitative Insights Into Microbial Ecology
LEfSe: Linear Discriminant Analysis Effect Size tool
PICRUSt: Phylogenetic Investigation of Communities by Reconstruction of Unobserved States software
ASVs: Amplification sequence variants
