# Supplementary material for: Psyllium supplementation is associated with changes in the fecal microbiota of horses
Source: BMC Res Notes. 2020 Sep 29;13:459. doi: 10.1186/s13104-020-05305-w (PMC7526151; doi:10.1186/s13104-020-05305-w)
Supplement: Supplementary file 1 — Additional file 1: Table S1. Details on horses from the study. Table S2. Yearling LEfSe Findings. Table S3. Adult LEfSe findings. Figure S1. Microbial DNA next-generation sequencing data analysis methodology. Figure S2. Analyses of diversity. [file 13104_2020_5305_MOESM1_ESM.pdf]

Psyllium Supplementation is Associated with Changes in the Fecal Microbiota of Horses

Michael J. Mienaltowski*, Ashley Belt, John D. Henderson, Tannah N. Boyd, Nicole Marter, Elizabeth A. Maga, Edward J. DePeters

Supplementary Material

Supplemental Tables:

Table S1. Details on horses from the study.

Horse ID	Group	Age (Yrs)	Sex	Breed
A	Yearling	1	Filly	Thoroughbred
B	Yearling	1	Filly	Quarterhorse/Paint Horse
C	Yearling	1	Colt	Azteca
D	Yearling	1	Colt	Azteca
E	Yearling	1	Colt	Quarterhorse
F	Yearling	1	Colt	Quarterhorse
201W	Adult	19	Mare	Quarterhorse
203W	Adult	22	Mare	Quarterhorse
208W	Adult	10	Mare	Hanoverian
23B	Adult	27	Mare	Quarterhorse
32B	Adult	21	Mare	Quarterhorse
344Y	Adult	30	Mare	Quarterhorse

Table S2. Yearling LEfSe Findings

Family	LEfSe LDA	Day	Wilcoxon p-value	Mean Abundance						
				Day 0	Day3	Day 7	Day 11	Day 14	Day 21	Day 28
Methylophilaceae	3.26	3	0.038	0.00%	***0.05%***	0.02%	0.00%	0.04%	0.02%	0.00%
Burkholderiaceae	2.98	3	0.011	0.00%	***0.14%***	0.01%	0.00%	0.09%	0.00%	0.00%
Saprospiraceae	3.16	14	0.030	0.00%	0.00%	0.01%	0.00%	***0.01%***	0.00%	0.00%
Neisseriaceae	3.62	21	0.003	0.10%	0.36%	0.07%	0.00%	0.37%	***0.60%***	0.00%
Fibrobacteraceae	4.28	28	0.016	1.69%	1.56%	2.08%	0.59%	1.04%	1.45%	***4.86%***
Paraprevotellaceae	4.13	28	0.020	2.64%	1.96%	3.25%	1.47%	1.72%	3.62%	***4.38%***

LEfSe LDA, Linear Discriminant Analysis Effect Size; Kruskal-Wallis Test $p < 0.05$ followed by one-against-all Wilcoxon

Table S3. Adult LEfSe Findings

Family	LEfSe LDA	Day	Wilcoxon p-value	Mean Abundance						
				Day 0	Day3	Day 7	Day 11	Day 14	Day 21	Day 28
Victivallaceae	2.97	21	0.033	0.00%	0.11%	0.02%	0.02%	0.01%	***1.16%***	0.02%
Methanobacteriaceae	3.51	28	0.023	0.09%	0.17%	0.49%	0.32%	0.15%	0.02%	***0.51%***
Methanocorpusculaceae	3.40	3	0.038	0.09%	***0.37%***	0.13%	0.16%	0.10%	0.02%	0.12%
Bacteroidaceae	3.82	0	0.045	***2.03%***	1.35%	1.31%	0.50%	1.40%	0.73%	0.75%
Moraxellaceae	3.71	7	0.009	0.15%	0.17%	***1.14%***	0.22%	0.28%	0.08%	0.57%

LEfSe LDA, Linear Discriminant Analysis Effect Size; Kruskal-Wallis Test $p < 0.05$ followed by one-against-all Wilcoxon

Supplemental Figures:

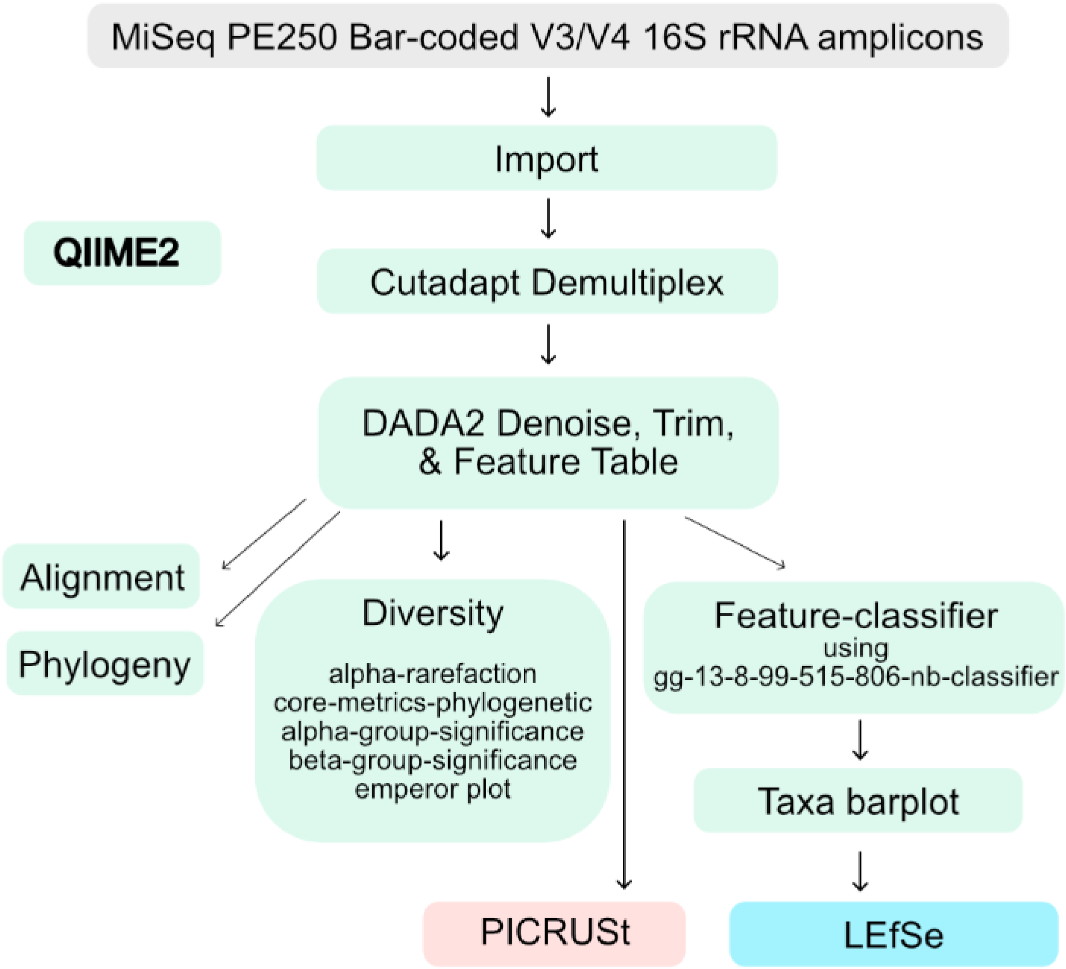

Figure S1. Microbial DNA next-generation sequencing data analysis methodology. Sequencing data were analyzed using QIIME2 (Quantitative Insights Into Microbial Ecology v2) found at https://qiime2.org/, LEfSe (Linear Discriminant of Effect Size) and PICRUSt (Phylogenetic Investigation of Communities by Reconstruction of Unobserved States), both found at: https://huttenhower.sph.harvard.edu/galaxy/.

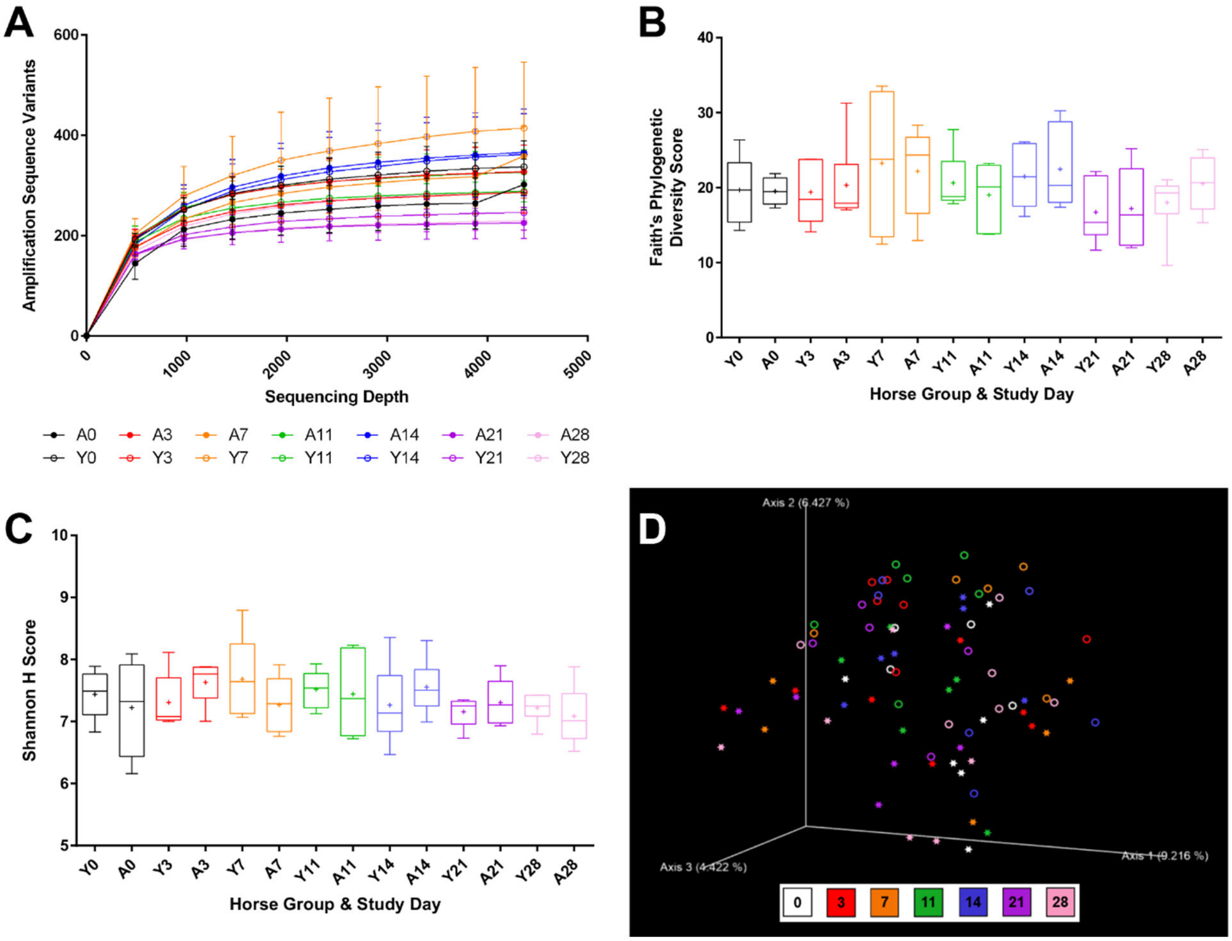

Figure S2. Analyses of Diversity. QIIME2 was used to examine diversity. Alpha and beta diversity of microbial populations among the samples were analyzed via: **(A)** Rarefaction Plot, **(B)** Faith's Phylogenetic Diversity Score, **(C)** Shannon H Score, and **(D)** Unifrac Unweighted Principal Component Analysis (PCA). In the Rarefaction Plot **(A)**, operational taxonomic units are plotted as means ± SEM. Faith's Phylogenetic Diversity **(B)** and Shannon H Score **(C)** are presented as box plots with boxes representing 25%-75% quartiles, lines within boxes representing medians, "+" representing mean scores, and upper and lower limits given as whiskers. "A" and "Y" represents adults and yearlings, respectively, in panels **(A)**, **(B)**, and **(C)**. **(D)** In the PCA, open circles depict adult mare samples and stars represent yearling samples.
